# Linoleic Acid and Linolenic Acid May Alleviate Heart Failure Through Aquaporin (AQP1) and Gut Microbiota

**DOI:** 10.3390/foods14203541

**Published:** 2025-10-17

**Authors:** Haotian Li, Jianqin Yang, Yubo Li, Yuewei Song, Qing Miao, Yanjun Liu

**Affiliations:** 1 Institute of Basic Theory of Traditional Chinese Medicine, China Academy of Chinese Medical Sciences, Beijing 100700, China; 20240931064@bucm.edu.cn (H.L.);; 2 Department of Digestive Disorders, Beijing Key Laboratory of Diagnosis and Treatment of Functional Gastrointestinal Diseases of Traditional Chinese Medicine, Beijing 100102, China; 3 School of Traditional Chinese Medicine, Beijing University of Chinese Medicine, Beijing 100029, China

**Keywords:** AQP1, linolenic acid, linoleic acid, chronic heart failure, floating wheat (*Fu Xiao Mai*), gut microbiota, *Triticum aestivum* L., wheat

## Abstract

Background: Chronic heart failure (CHF) is a major cause of morbidity and mortality worldwide, with limited therapeutic options. Floating wheat (Fu Xiao Mai), used in traditional Chinese medicine for CHF, contains linoleic acid (LA) and α-linolenic acid (ALA) as major bioactive components, but their therapeutic mechanisms remain unclear. Objective: this study aimed to investigate the cardioprotective effects of LA and ALA in CHF, focusing on their interactions with aquaporin-1 (AQP1) and gut microbiota. Methods: LA and ALA were identified in floating wheat via LC-MS/MS. Molecular docking and dynamics simulations assessed their binding to AQP1. In vivo studies used C57BL/6 and AQP1^−^/^−^ mice with isoproterenol-induced CHF. Cardiac function was assessed through echocardiography; myocardial ultrastructure through transmission electron microscopy (TEM); inflammatory markers (TNF-α, NO, VEGF, VCAM-1) through ELISA; and gut microbiota through 16S rRNA sequencing. Results: Molecular docking revealed a strong binding affinity of LA and ALA to AQP1, with binding energies of −8.532 kcal/mol and −8.835 kcal/mol, respectively. In C57 mice, LA and ALA administration significantly improved cardiac function (*p* < 0.05, the high-dose group compared to the model group) while reducing myocardial edema. They also downregulated AQP1 expression and decreased levels of inflammatory markers (*p* < 0.05, the high-dose group compared to the model group). These functional improvements were significantly attenuated in AQP1^−^/^−^ mice. However, the reduction in inflammatory markers persisted, indicating AQP1-independent anti-inflammatory effects. Furthermore, high-dose LA/ALA treatment in AQP1^−^/^−^ mice markedly altered gut microbiota. Conclusion: LA and ALA alleviate CHF through an AQP1-dependent reduction in myocardial edema and AQP1-independent anti-inflammatory and gut microbiota-modulating effects. These findings highlight their potential as a multi-target therapeutic complex for CHF.

## 1. Introduction

*Fu Xiao Mai*, also known as floating wheat, refers to the dry, light, and thin fruit of the gramineous plant *Triticum aestivum* L., which earns its name from its ability to float on water. In contrast, common wheat (*Triticum aestivum* L.) denotes the dry, mature, and heavy fruit that sinks in water. Traditional Chinese medicine attributes certain efficacy of floating wheat to chronic heart failure (CHF) and myocardial edema [1], whereas common wheat is not considered therapeutic for these conditions but is used for restlessness and insomnia [2]. We hypothesize that the differential therapeutic effects between these two types of wheat are likely attributable to distinct bioactive components. Previous studies have identified linoleic acid and linolenic acid as the major differential compounds [3]; however, their mechanisms of action remain inadequately investigated.

CHF results from various cardiac diseases leading to abnormal heart structure and function, impairing ventricular filling or ejection capacity. As the terminal stage of most cardiovascular diseases, CHF represents a major health burden in the middle-aged and elderly population, with high mortality and rehospitalization rates. Data indicates that, with an increasing life expectancy and the growing prevalence of obesity and diabetes, the burden of CHF continues to rise. The prevalence of heart failure in the adult population of developed countries is approximately 2.0%, exceeding 10% among those over 70 years of age, with more than 500,000 new cases reported annually [4]. Thus, CHF represents both the final frontier and the most critical challenge in contemporary cardiovascular disease management.

The cornerstone of CHF pharmacotherapy involves a multimodal approach targeting distinct pathophysiological mechanisms: (1) diuretics to manage volume overload by enhancing sodium excretion; (2) neurohormonal modulators, including angiotensin-converting enzyme inhibitors (ACEIs), angiotensin receptor blockers (ARBs), and mineralocorticoid receptor antagonists (MRAs), to inhibit the renin–angiotensin–aldosterone system (RAAS); (3) β-adrenergic receptor antagonists to modulate sympathetic nervous system activity; and (4) emerging therapies targeting myocardial energetics and cellular function [5]. However, clinical efficacy remains suboptimal, and many patients continue to experience unresolved symptoms. There is a clear lack of drugs capable of preventing heart failure or modifying disease progression, highlighting an urgent need for novel treatment strategies.

Accumulating evidence suggests that ventricular remodeling serves as a common pathway in the progression of various structural heart diseases to CHF. This process is characterized by abnormal cardiac geometry, cardiomyocyte hypertrophy with functional impairment and a reduced cell number, and increased cardiac stromal cells, accompanied by declining cardiac function. Physicochemical factors, pathological stimuli, autoimmunity, inflammatory reactions, and other factors can initiate ventricular remodeling. Identifying strategies to pharmacologically inhibit ventricular remodeling, delay heart failure progression, and improve prognoses carries significant clinical and societal benefits.

Aquaporins (AQPs), a conserved family of transmembrane channel proteins, are ubiquitously expressed in human and murine organ systems. These tetrameric assemblies facilitate selective water transport through their hourglass-shaped pores, critically regulating fluid homeostasis, cellular osmoregulation, and epithelial secretion processes. Among the AQP family, AQP1 is the most abundantly expressed aquaporin in human and mouse myocardial tissues, primarily localized on vascular endothelial cells and cardiomyocyte membranes, where it maintains water balance by mediating transmembrane water transport. Aberrant AQP1 expression can induce cardiomyocyte edema, increase microvascular permeability, and disrupt cardiomyocyte function. AQP1 participates in multiple pathological processes in heart failure, including myocardial interstitial edema, inflammation-driven angiogenesis, and tissue repair. Myocardial edema itself exerts substantial detrimental effects on cardiac tissue; edema-induced compression can impair microcirculation, compromise blood and oxygen supply, and create a vicious cycle that exacerbates ventricular remodeling and worsens pathological processes such as left ventricular diastolic dysfunction and increased cardiac afterload.

Growing evidence suggests that dietary polyunsaturated fatty acids (PUFAs), particularly the essential fatty acids linoleic acid (LA, an omega-6 PUFA) and α-linolenic acid (ALA, an omega-3 PUFA), may play a beneficial role in modulating the progression and management of CHF. ALA, as a plant-derived omega-3 PUFA, has been associated with improved cardiovascular health. Research indicates it can help reduce atherogenic lipids and lipoproteins (like total cholesterol, LDL, and triglycerides), lower blood pressure, and curb inflammation [6]. Given that inflammation is a key driver of cardiac remodeling in CHF, the anti-inflammatory properties of ALA are of particular interest. LA also contributes to these regulatory processes, and both fatty acids can influence the composition of the cardiac lipidome, which is crucial for maintaining myocardial energy metabolism and structural integrity [7].

Current research increasingly indicates that ischemia and hypoxia can induce cardiomyocyte edema during heart failure development, accompanied by aberrant AQP1 protein distribution in necrotic tissues and vascular endothelial inflammatory responses, leading to disorganized myocardial tissue structure [8]. Notably, AQP1 significantly influences core pathophysiological processes in heart failure, such as left ventricular diastolic dysfunction and cardiac afterload, through its involvement in key pathological mechanisms, including myocardial interstitial edema, inflammation-mediated angiogenesis, and tissue repair [9]. This study investigates the therapeutic mechanisms of LA and ALA in CHF through the regulation of AQP1.

## 2. Materials and Methods

### 2.1. Molecular Docking and Molecular Dynamics Simulation

The three-dimensional structure of the target protein was obtained from the RCSB Protein Data Bank (https://www.rcsb.org/, accessed on 1 September 2025) [10]. Molecular docking, including protein and ligand preparation and interaction analysis, was performed with AutoDockTools 1.5.7. Subsequently, molecular dynamics (MD) simulations were conducted using GROMACS 2022.3 [11]. The topology parameters for small molecules were generated and incorporated into the simulation system [12]. The simulations were performed under constant temperature (310 K) and pressure (1 bar) conditions for 100 ns, employing a 2 fs integration time step over 5,000,000 steps. The Amber99sb-ildn force field and the Tip3p water model were used throughout the simulations.

### 2.2. Animal Experiments

#### 2.2.1. Construction of AQP1 Knockout Mice

The information concerning the construction of AQP1 Knockout Mice in this study is the same as that presented in our previous article [13].

#### 2.2.2. Experimental Grouping

C57 mice are the genetic background of knockout mice as a negative control. Knockout mice and C57 mice were intraperitoneally injected with isoproterenol (ISO) to establish a mouse heart failure model, and the ISO dose was 30 mg/kg per day for 21 days. After the model, echocardiography was performed, and the mice that were successfully modeled were divided into a model group, a positive group, a high-dose group, a medium-dose group, and a low-dose group, with 10 mice in each group, for a total of 6 groups. The positive drug captopril was administered via gavage at 10 mg/(kg·d), with 0.1 mL of gavage once a day, and the other groups were given the same volume of normal saline; high-dose-group mice were fed an ad lib diet of standard chow with 200 mg/(kg·d) linoleic acid and linolenic acid, while medium-dose-group mice were fed an ad lib diet of standard chow with 100 mg/(kg·d) linoleic acid and linolenic acid. :ow-dose-group mice were fed an ad lib diet of standard chow with 50 mg/(kg·d) linoleic acid and linolenic acid. Linoleic acid and linolenic acid were brought from Solarbio with the IDs of Cat: SL8520 and Cat: SL8530.

### 2.3. Detection of Function Changes in CHF Mice

#### 2.3.1. Transmission Electron Microscopy Observation

Take 3~4 myocardial tissue specimens (1 mm^3^) and immediately put them into phosphate-buffered acid (PBS) configuration in 2.5% glutaraldehyde for pre-fixation. After rinsing with PBS rinse solution, osmium acid is fixed. Administer ethanol and acetone gradient dehydration, epoxy resin immersion overnight, embedding, sectioning, staining, and transmission electron microscopy to observe morphological changes.

#### 2.3.2. Elisa Detection

Following initial coating with anti-NT-proBNP, NO, VCAM, VEGF, and TNF-α antibodies, the assay procedure was conducted sequentially: (1) transfer of samples and standards into designated wells, (2) introduction of horseradish peroxidase-conjugated secondary antibodies, and (3) incubation (37 °C, 60 min), followed by extensive washing. Chromogenic development was initiated via a TMB substrate addition, wherein enzymatic catalysis induced sequential color transition (colorless → blue → yellow) upon acid termination. The resultant color intensity (measured at 450 nm via microplate spectrophotometry) demonstrated direct proportionality to the NT-proBNP concentration, enabling quantitative determination through standard curve interpolation.

#### 2.3.3. Western Blotting, RT-PCR, Echocardiography, and Sequencing of Mouse 16S rDNA Gut Microbiota

The Western blotting, RT-PCR, and Sequencing of Mouse 16S rDNA Gut Microbiota experimental procedure was consistent with our previous study [13]. The antibodies used were as follows. VEGF: (1: 10,000, GB11034B, Servicebio, Wuhan, China); β-catenin: (1: 10,000, GB12015, Servicebio); GAPDH: (1: 20,000, GB15002, Servicebio). Primer sequence: VEGF: (GAGCGTTCACTGTGAGCCTTGT TTAACTCAAGCTGCCTCGCCT) β-catenin: (TGCAGTTGCTTTATTCTCCCAT TGTTGCCACGCCTTCATTC).

### 2.4. LC-MS/MS Analysis of Fu Xiao Mai Components

An aliquot (150 μL) of the “*fu xiao mai*” (samples came from the Wangjing Hospital in accordance with standard clinical protocols) was mixed with 150 μL of 70% methanol meeting internal standards (2 μg/mL). After vortexing (1 min) and ice-bath ultrasonic extraction (60 min), the mixture was centrifuged (12,000 rpm, 4 °C, 10 min). The supernatant (200 μL) was analyzed using UPLC-MS/MS with an ACQUITY UPLC HSS T3 column (45 °C) and a gradient of 0.1% formic acid in water (A) and acetonitrile (B) at 0.35 mL/min. Mass detection was performed on a Thermo Orbitrap QE HF mass spectrometer in DDA mode. Data was processed using XCMS V3.7.1, with compound identification against the LuMet-TCM database (score ≥ 40).

### 2.5. Statistical Analysis

All quantitative variables are presented as means ± standard deviations (SDs). Data processing was conducted using SPSS Statistics (version 21.0, IBM Corp., Armonk, NY, USA). For intergroup comparisons, a one-way analysis of variance (ANOVA) was performed, followed by post hoc analyses: for data with homogeneous variance, the LSD post hoc test was used; for data with heterogeneous variance, Dunnett’s T3 test was applied. A probability value of *p* < 0.05 was considered statistically significant.

## 3. Results

### 3.1. Molecular Dynamics Simulation and LC&MS Results

**Figure 1:**
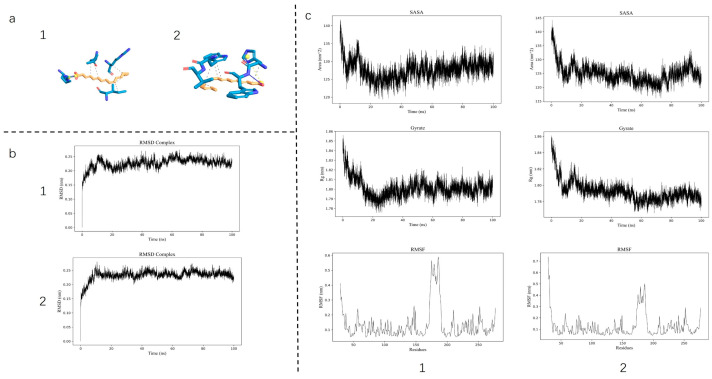
Molecular docking visualization and molecular dynamics simulation results with 1: linoleic acid and AQP1 and 2: linolenic acid and AQP1; (**a**) visualization of molecular docking; (**b**) RMSD; (**c**) from top to bottom, SASA, Rg, RMSF.

**Figure 2:**
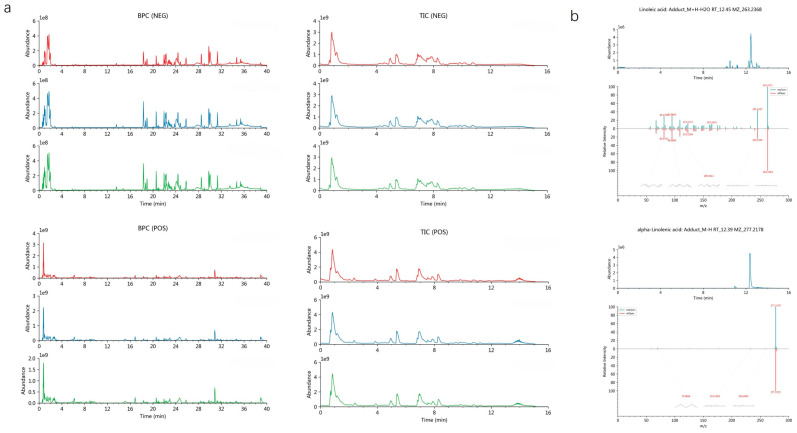
Analysis of LA&ALA via LC-MS/MS. (**a**) Representative base peak chromatogram (BPC) and total ion chromatogram (TIC) of the Fu Xiao Mai analyzed. (**b**) Mass spectrum of LA&ALA.

Molecular docking revealed a substantial binding affinity between the core active compounds and the target protein, with calculated binding energies of −8.532 kcal/mol for LA and −8.835 kcal/mol for ALA. MD further demonstrated the stability of these complexes, as indicated by a consistently low root-mean-square deviation (RMSD), a decrease in root-mean-square fluctuation (RMSF) of the ligand–protein complex, and a reduction in both the solvent-accessible surface area (SASA) and the radius of gyration (Rg)—collectively suggesting enhanced structural compactness upon ligand binding (Figure 1). These computational results confirm a stable interaction between AQP1 and the two fatty acids. Furthermore, to robustly confirm prior findings, our LC-MS/MS analysis unequivocally verified the presence of both LA and ALA in floating wheat (Figure 2), thereby identifying them as the principal bioactive constituents. Together, these findings corroborate the proposition that the cardioprotective effects of LA and ALA observed in vivo may be mediated, at least in part, through the direct regulation of AQP1.

### 3.2. Establishment and Confirmation of AQP1 Knockout Disease Model and 24 h Urine Output

**Figure 3:**
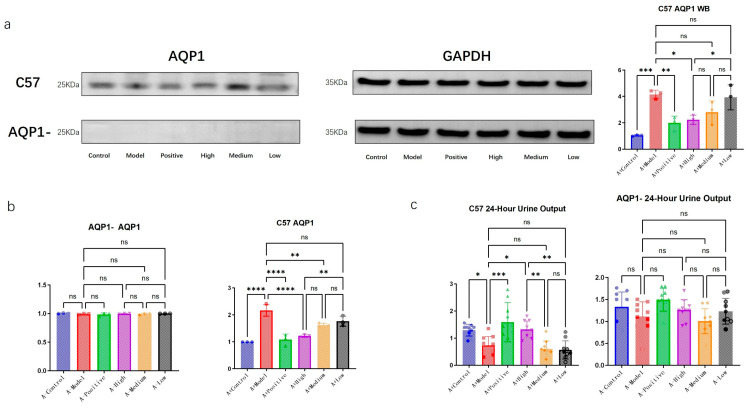
Validation of AQP1 knockout and its functional impact on urine output. (**a**) Western blot analysis of AQP1 protein expression in cardiac tissues of C57 and AQP1^−^/^−^ mice across the indicated groups. GAPDH was used as a loading control. (**b**) mRNA expression levels of AQP1 determined by RT-qPCR. (**c**) Measurement of 24 h urine output in different groups. Data are presented as means ± SEMs (n = 3 for a,b; n = 6 for c). * *p* < 0.05, ** *p* < 0.01, *** *p* < 0.001, **** *p* < 0.0001; ns, not significant (one-way ANOVA with post hoc test).

We performed PCR and WB analyses to further validate the successful knockout of the target gene. As shown in Figure 3, no AQP1-related protein was detected in the AQP1-knockout group with WB, while no significant difference in each group was observed at the mRNA level through PCR, confirming the successful establishment of the genetic knockout model.

In C57 mice, the expression of AQP1 protein increased with the progression of heart failure (HF), indicating a correlation between AQP1 expression and heart failure. Among the treatment groups in C57 mice, all administered doses led to a reduction in AQP1 expression, with consistent trends observed in both PCR and WB results.

Furthermore, with the induction of heart failure, a significant decrease in urine output was observed in the C57 mouse group. Within the treatment groups, the urine volume gradually decreased with diminishing doses. In contrast, no significant differences in urine output were detected among the AQP1-knockout groups.

### 3.3. LA and ALA Alleviate Heart Failure Symptoms and Improve Ventricular Remodeling, Both of Which May Be Highly Correlated with AQP1

**Figure 4:**
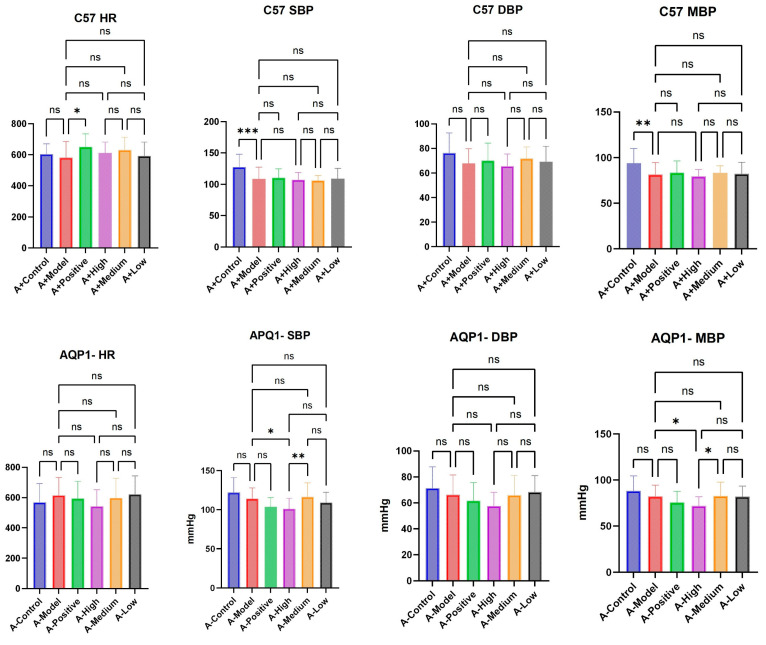
Statistical diagram of C57 and AQP1^−^/^−^’s HR (heart rate), SBP (systolic blood pressure), DBP (diastolic blood pressure), MBP (mean blood pressure). Data are presented as means ± SEMs (n = 27 per group, one-way ANOVA was used, ns *p* > 0.05, * *p* < 0.05, ** *p* < 0.01, *** *p* < 0.001 ).

In the C57 group, the establishment of a heart failure model resulted in significant differences in SBP (systolic blood pressure) and MBP (mean blood pressure), but in the AQP1 knockout group, there was no difference between the heart failure model and the normal group. The treatment groups of both groups of mice did not have a significant effect on HR (heart rate), SBP, MBP, or DBP (diastolic blood pressure) (Figure 4).

**Figure 5:**
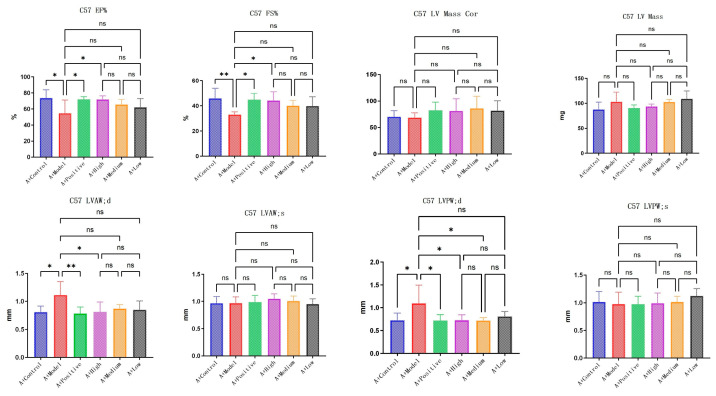
Statistical diagram of C57’s echocardiographic. Data are presented as means ± SEMs (n = 6 per group, one-way ANOVA was used, ns *p* > 0.05, * *p* < 0.05, ** *p* < 0.01  ).

**Figure 6:**
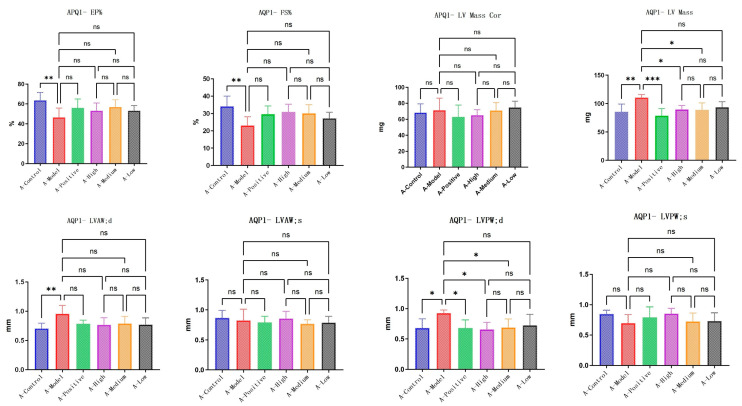
Statistical diagram of AQP1^−^/^−^’s echocardiographic. Data are presented as means ± SEMs (n = 6 per group, one-way ANOVA was used, ns *p* > 0.05, * *p* < 0.05, ** *p* < 0.01, *** *p* < 0.001 ).

LA&ALA effectively alleviates symptoms of chronic heart failure and improves ventricular remodeling, a pharmacological effect potentially associated with the AQP1 gene. In C57 mice, parameters including EF% (ejection fraction), FS% (fractional shortening), LVAW;d (left ventricular anterior wall thickness at end-diastole), and LVPW;d (left ventricular posterior wall thickness at end-diastole) showed significant differences (*p* < 0.05) in the model group compared to the normal group, which is consistent with characteristic changes in heart failure. Compared to the model group, the high-dose LA&ALA group exhibited significant improvements in EF%, FS%, LVAW;d, and LVPW;d, with statistically significant differences (*p* < 0.05) (Figure 5). In AQP1^−^/^−^ mice, significant differences were observed in EF%, FS%, and LV Mass (left ventricular mass) between the model group and the normal group. However, in the high-dose LA&ALA group compared to the model group, the original improvement in ventricular remodeling was abolished, with no significant enhancement in EF%, FS%, or LVAW;d (*p* > 0.05). It is also noteworthy that AQP1 knockout led to a significant increase in LV Mass, while no notable difference was found in LV Mass Cor (left ventricular mass corrected). These results suggest that the heart failure model exacerbates myocardial fluid retention and that AQP1 is highly involved in this process, consistent with previous studies. Furthermore, AQP1 knockout did not appear to affect the improvement in LVPW;d, as a significant difference remained between the treatment and model groups (*p* < 0.05). In contrast, the improvement in LVAW;d was eliminated (*p* > 0.05) (Figure 6).

### 3.4. The Improvements in Immune Response and Angiogenesis Following LA and ALA Treatment Are Mediated by a Mechanism Independent of AQP1

**Figure 7:**
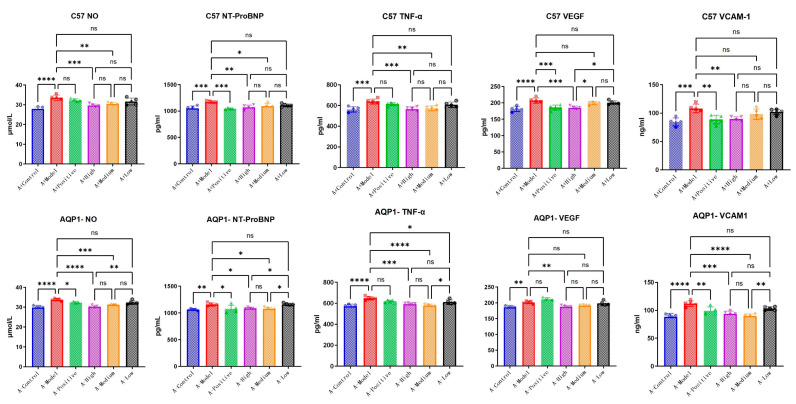
Concentrations of NO, NT-ProBNP, TNF-α, VEGF, and VCAM in serum measured via ELISA. Data are presented as means ± SEMs (n = 5 per group, one-way ANOVA was used, ns *p* > 0.05, * *p* < 0.05, ** *p* < 0.01, *** *p* < 0.001, **** *p* < 0.0001).

To further elucidate the specific mechanisms through which LA and ALA improve heart failure symptoms, we conducted exploration testing on several candidate pathways provided in the literature, including inflammatory response (TNF-α, VCAM-1) [14] and vasodilation (VEGF) [15]. In C57 mice, NT-ProBNP expression was significantly elevated in the model group compared with the normal group (*p* < 0.05). Treatment with high-dose LA&ALA significantly attenuated the expression of NT-ProBNP, NO, VCAM, VEGF, and TNF-α compared with the model group (*p* < 0.05), indicating an amelioration in heart failure symptoms. In AQP1^−^/^−^ mice, although NT-ProBNP remained significantly higher in the model group than in the normal control group (*p* < 0.05). No significant difference was observed between the LA&ALA treatment group and the model group. This shows that the effect of LA&ALA on heart failure—particularly in reducing NT-ProBNP—is highly dependent on AQP1 expression. Interestingly, LA&ALA still significantly modulated NO, VCAM, VEGF, and TNF-α levels, even in the absence of AQP1 (Figure 7), indicating that these effects may operate through AQP1-independent pathways.

The ELISA results confirm that LA and ALA not only improve cardiac function in chronic heart failure but also exert vascular protective effects by reducing inflammatory cytokines, inhibiting NO overproduction, and decreasing serum levels of VEGF and VCAM-1. Importantly, the pharmacological reductions in VEGF, VCAM-1, and NO appear to be independent of AQP1, suggesting that LA&ALA may act through multiple parallel mechanisms. For instance, its anti-inflammatory and endothelium-stabilizing effects may involve alternative signaling pathways such as NF-κB inhibition or PI3K/Akt activation. These findings highlight the complex and multifaceted mechanism of action underlying LA and ALA’s cardioprotective effects.

### 3.5. The Efficacy of LA and ALA in Improving Ventricular Myocardial Edema Is Contingent on AQP1

To further elucidate the specific mechanisms through which LA&ALA influences ventricular remodeling, we investigated several potential pathways, focusing on VEGF—a key factor in promoting angiogenesis [16]—and β-catenin, which is involved in regulating cell adhesion and mitigating myocardial edema [17].

**Figure 8:**
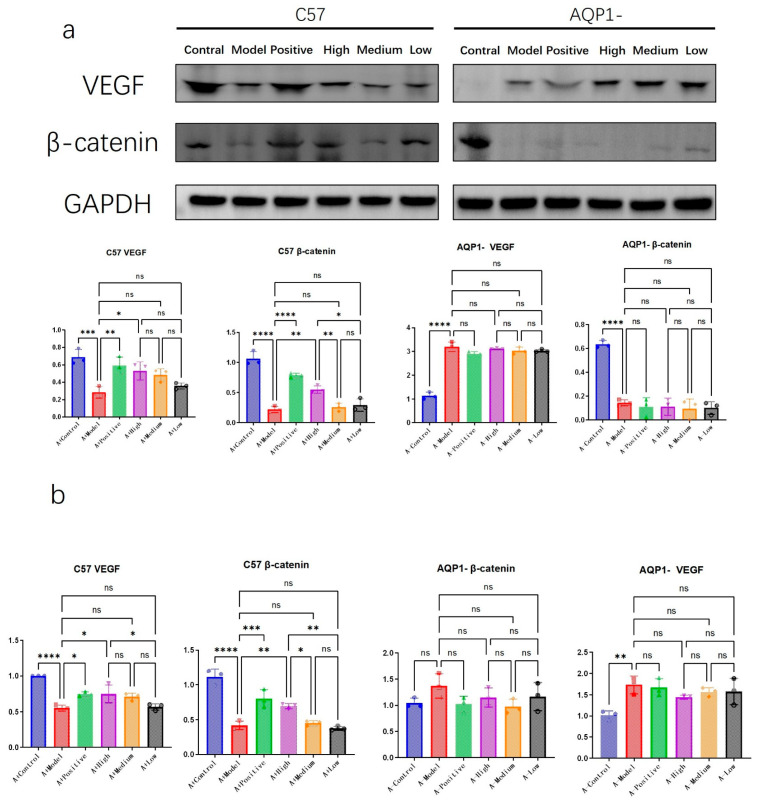
LA and ALA can improve ventricular myocardial edema and depend on AQP1 (**a**) Western blot analysis of VEGF and β-catenin protein levels. Densitometric quantification of protein bands normalized to the loading control is presented as mean ± SEM. (n = 3 per group, one-way ANOVA was used, ns *p* > 0.05, * *p* < 0.05, ** *p* < 0.01, *** *p* < 0.001, **** *p* < 0.0001). (**b**) VEGF and β-catenin copy number determined via RT-PCR. (n = 3 per group, one-way ANOVA was used, ns *p* > 0.05, * *p* < 0.05, ** *p* < 0.01, *** *p* < 0.001, **** *p* < 0.0001).

Western blot and RT-PCR results suggested a possible interaction between β-catenin and AQP1 (Figure 8). In AQP1^−^/^−^ mice, expression of β-catenin, a critical adherent junction protein involved in Wnt signaling and tissue integrity, was significantly reduced. Among C57 groups, high-dose LA&ALA treatment resulted in elevated β-catenin expression (*p* < 0.05). Given the absence of such improvement in AQP1^−^/^−^ mice, this effect may be associated with the amelioration of interstitial ventricular edema.

In contrast, VEGF expression trends appeared independent of AQP1 knockout. VEGF—a major mediator of angiogenesis and vascular permeability—may be activated via LA&ALA to promote vascular regeneration and alleviate heart failure symptoms, indicating that this pharmacological effect operates through an AQP1-independent pathway.

**Figure 9:**
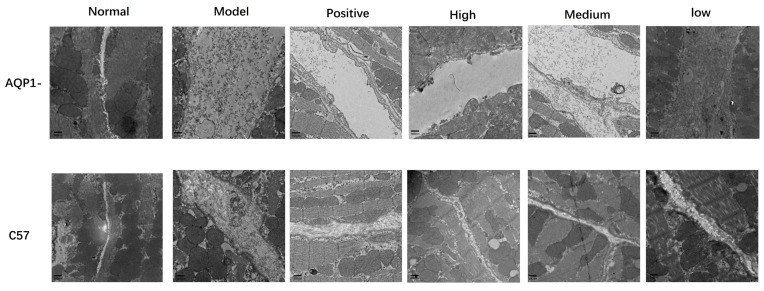
Representative TEM images in the myocardial tissue. Scale bars = 500 nm.

To further determine whether LA and ALA ameliorate myocardial interstitial edema, we employed TEM to examine ultrastructural changes in the myocardial tissue. TEM analysis revealed distinct differences across groups. In normal mice, interstitial collagen fibers showed well-organized, parallel or reticular arrangements with uniform spacing. In contrast, the model group exhibited markedly widened interstitial spacing, prominent electron-lucent zones (indicative of fluid accumulation), and disrupted tight junctions. LA and ALA treatment substantially attenuated these abnormalities, resulting in narrower collagen spacing, reduced edema, and restored junctional integrity (Figure 9).

### 3.6. High-Dose LA and ALA Treatment May Affect Gut Microbiota

**Figure 10:**
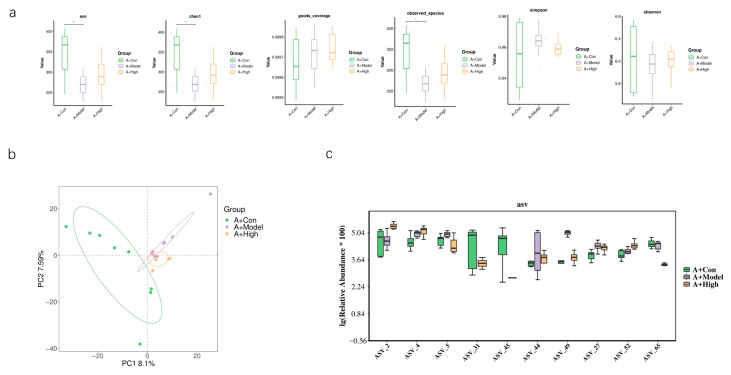
The gut microbiota composition in C57 mice. A + Con is a control group of C57 mice; A + Model is a CHF group of C57 mice; and A + High is the high-dose LA&ALA group of C57 mice. (**a**) The alpha diversity analysis of gut microbiota in wild-type mice; (**b**) the beta diversity analysis (PCA) of gut microbiota composition. (**c**) The box plot of the top 10 different ASV values. (n = 8 *: <0.05 ).

**Figure 11:**
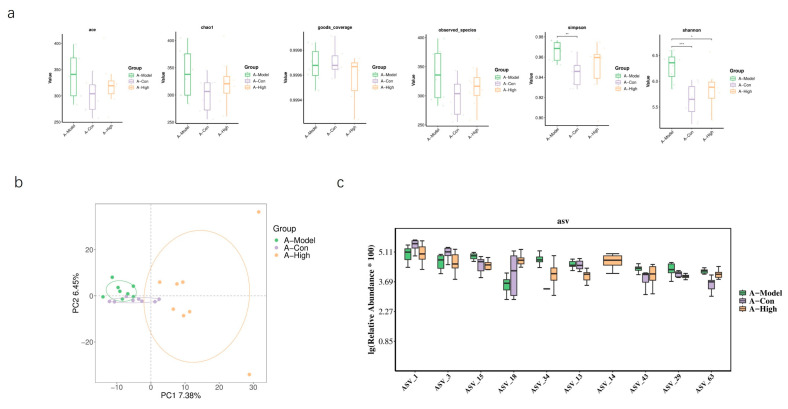
The gut microbiota composition in AQP1^−^/^−^ mice. A-Con is a control group of AQP1^−^/^−^ mice; A-Model is a CHF group of AQP1^−^/^−^ mice; and A-High is the high-dose LA&ALA group of AQP1^−^/^−^ mice. (**a**) The alpha diversity analysis of gut microbiota in AQP1^−^/^−^ mice; (**b**) the beta diversity analysis (PCA) of gut microbiota composition. (**c**) The box plot of the top 10 different asv. (n = 8 *: <0.05; **: <0.01; ***: <0.001).

**Figure 12:**
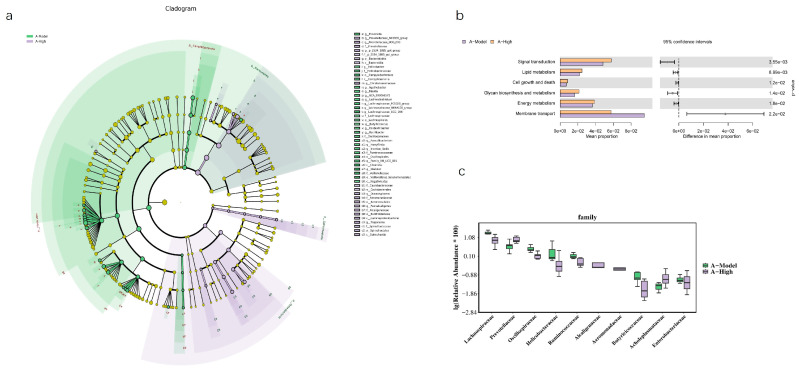
Different gut microbiota analysis. A-Con is a control group of AQP1^−^/^−^ mice; A-Model is a CHF group of AQP1^−^/^−^ mice; and A-High is the high-dose LA and ALA group of wild-type AQP1^−^/^−^ mice. (**a**) The LEFSe analysis; (**b**) the box plot of the top 10 KEGG enrichment (n = 6, T-test). (**c**) The boxmap of the top 15 different families’ gut microbiota between A-Model and A-High.

Based on our previous findings regarding the association between the heart failure model and AQP1 knockout, we selected the high-dose treatment group, which exhibited the most pronounced therapeutic effects, for gut microbiota sequencing. In normal C57 mice, alpha diversity analysis showed no significant differences between the treatment and model groups. However, PCA revealed a limited overlap between the groups, suggesting considerable divergence in microbial composition (Figure 10). In AQP1^−^/^−^ mice, alpha diversity indices indicated a significant difference in microbial community structure between the high-dose group and the model group. Differential abundance analysis highlighted significant changes in bacteria belonging to the families *Lachnospiraceae* and *Prevotellaceae*. Consistent with this, PCA showed clear separation between these groups with minimal overlap in ordination space (Figure 11). An LEFSe analysis was performed to identify specific taxes contributing to these differences (Figure 12). To further elucidate the potential functional implications of the altered gut microbiota, we performed functional prediction analysis using Tax4fun2 based on the 16S rRNA sequencing data [18]. The predicted functional profiles were mapped to the KEGG pathway database. We observed significant differences in several microbial metabolic pathways between the A-High and A-Model groups (Figure 12). Specifically, the high-dose LA&ALA treatment group exhibited a predicted enrichment in pathways related to lipid metabolism, energy metabolism, glycan biosynthesis, and metabolism (*p* < 0.05). Conversely, pathways associated with membrane transport were significantly attenuated in the A-High group compared to the A-Model group (*p* < 0.01). These predictions suggest that the modulation of gut microbiota via LA&ALA, particularly the increase in *Prevotellaceae_NK3B31_group* and the decrease in *Lachnospiraceae_NK4A136_group*, may functionally contribute to ameliorating heart failure by enhancing beneficial metabolite production and reducing systemic inflammation.

## 4. Discussion

The application of novel tools and methodologies is opening new avenues for the scientific understanding of traditional medicine, paving the way for a more sophisticated understanding of its underlying principles [19,20,21,22]. Our findings demonstrate that LA and ALA, the principal bioactive components of floating wheat, exert protective effects against CHF through a multi-mechanistic framework that intricately links direct molecular targeting, systemic anti-inflammation, and gut microbiota modulation.

The initial premise of this study was that the therapeutic effects of LA and ALA might be mediated through direct interaction with AQP1. Our computational results from molecular docking and dynamics simulations provided a solid structural basis for this hypothesis. The strong binding affinities and the stable, compact ligand–protein complexes, as evidenced by low RMSD, RMSF, SASA, and Rg values, confirmed that both LA and ALA can physically interact with AQP1. This in silico prediction was functionally validated in vivo, as the cardioprotective effects—specifically the improvement in ventricular remodeling parameters (EF%, FS%, LVAW;d) and the reduction in myocardial edema and NT-proBNP—were profoundly attenuated in AQP1^−^/^−^ mice. This compelling synergy between computation and experiment establishes that the LA/ALA–AQP1 interaction is a crucial initial event that drives the alleviation of AQP1-mediated myocardial water imbalance.

Interestingly, not all beneficial effects were dependent on AQP1. The reduction in key inflammatory and vascular markers (TNF-α, VCAM-1, VEGF, NO) persisted in AQP1^−^/^−^ mice, revealing the existence of parallel, AQP1-independent pathways. This prompted us to investigate other systemic mechanisms, leading to the discovery of a significant role for the gut microbiota. High-dose LA/ALA treatment induced substantial shifts in the microbial community in AQP1^−^/^−^ mice, notably increasing *Prevotellaceae* and decreasing *Lachnospiraceae*. Functional prediction analysis further suggested that this altered microbiota profile was associated with enhanced lipid and energy metabolism. We propose that this gut microbiota remodeling serves as a complementary, AQP1-independent arm of LA/ALA’s action. The microbiota-derived metabolites (e.g., short-chain fatty acids or other anti-inflammatory molecules) could potentially circulate systemically to exert the observed anti-inflammatory and vascular protective effects, thereby explaining the persistence of these benefits even in the absence of AQP1 [8,23].

In conclusion, this study unveils a cohesive mechanistic model for LA and ALA in alleviating CHF: the fatty acids may directly engage AQP1, as predicted through computational modeling and confirmed in vivo, to combat the core pathology of myocardial edema. Concurrently, they reprogram the gut microbiota, which in turn contributes to the systemic mitigation of inflammation and improvement of metabolic function through a separate pathway. This dual-track mechanism—encompassing both a targeted, AQP1-centric approach and a broader, microbiota-mediated systemic strategy—highlights the unique multi-target potential of LA and ALA as a novel therapeutic complex for CHF. Future research should focus on validating the causal role of the specific microbial taxa identified and elucidating the precise metabolite signals that bridge the gut–heart axis in this context [24].

## 5. Conclusions

In conclusion, this study demonstrates that LA and ALA, derived from Fu Xiao Mai, exert protective effects against chronic heart failure through multi-mechanistic pathways. The combined administration of LA and ALA significantly improved cardiac function, reduced myocardial edema, and attenuated ventricular remodeling, largely through the modulation of AQP1 expression. Notably, these effects were markedly diminished in AQP1^−^/^−^ mice, underscoring the essential role of AQP1 in mediating the cardioprotective actions of these fatty acids. Additionally, LA and ALA independently reduced inflammatory markers and modulated gut microbiota composition, suggesting complementary non-AQP1-dependent mechanisms. These findings highlight the potential of LA and ALA as a novel therapeutic complex for CHF treatment and provide a robust foundation for further preclinical and clinical investigations.

## Data Availability

The original contributions presented in this study are included in the article. Further inquiries can be directed to the corresponding authors.
